# Case Report: *Mycoplasma pneumoniae*-induced rash and mucositis in a child

**DOI:** 10.3389/fmed.2025.1607970

**Published:** 2025-07-21

**Authors:** Xiaoying Mo, Meihua Wang, Xue Jiang, Li Jin

**Affiliations:** ^1^Department of Infection, Nantong Third People's Hospital, Affiliated Nantong Hospital 3 of Nantong University, Nantong, Jiangsu, China; ^2^Department of Emergency, Nantong Third People's Hospital, Affiliated Nantong Hospital 3 of Nantong University, Nantong, Jiangsu, China; ^3^Department of Emergency Medicine, The First Affiliated Hospital of Soochow University, Suzhou, Jiangsu, China

**Keywords:** *Mycoplasma pneumoniae*, rash and mucositis, MIRM, children, case report

## Abstract

**Rationale:**

*Mycoplasma pneumoniae* (MP) is a common pathogen responsible for pediatric community-acquired pneumonia. MP infection can also induce extrapulmonary manifestations, including mucocutaneous eruptions. *Mycoplasma pneumoniae*-induced rash and mucositis (MIRM) is a relatively newly described entity distinct from erythema multiforme (EM), Stevens-Johnson syndrome (SJS), and toxic epidermal necrolysis (TEN). However, MIRM remains underrecognized, with limited cases reported in China.

**Patient concerns:**

A previously healthy 10-year-old boy presented with fever, cough, severe mucositis (oral and ocular involvement), and disseminated rash for 5 days.

**Diagnoses:**

The patient was diagnosed with *Mycoplasma pneumoniae*-induced rash and mucositis (MIRM), confirmed by clinical presentation and laboratory detection of MP nucleic acid.

**Interventions:**

The treatment regimen for the patient included intravenous azithromycin, methylprednisolone at a dosage of 1 mg/kg/day, intravenous immunoglobulin (IVIG), vitamin C, oral antihistamines, topical treatments for mucosal care, ophthalmic ointment, and calamine lotion. After 1 week, due to a persistent cough and a positive *Mycoplasma pneumoniae* (MP) nucleic acid test, the patient’s treatment was transitioned to oral doxycycline.

**Outcomes:**

The patient experienced significant clinical improvement after 14 days, with resolution of rash, mucosal lesions, and cough. Follow-up tests confirmed clearance of MP nucleic acid and normalization of inflammatory markers.

**Lessons:**

MIRM is a distinct mucocutaneous entity associated with MP infection. Pediatricians should recognize its clinical presentation early to provide prompt targeted therapy and supportive care, thus preventing complications.

## Introduction

*Mycoplasma pneumoniae* (MP) is a frequent cause of respiratory tract infections in children (1). Up to 25% of affected children develop extrapulmonary complications, including mucocutaneous manifestations such as *Mycoplasma pneumoniae*-induced rash and mucositis (MIRM) (1–4). Recently, MIRM has been recognized as a distinct clinical entity, differentiated from erythema multiforme (EM), Stevens-Johnson syndrome (SJS), and toxic epidermal necrolysis (TEN), featuring prominent mucosal involvement with limited skin lesions (4, 5). Due to its rarity and recent recognition, MIRM remains underdiagnosed and understudied, especially in China (6). Here, we report a pediatric case of MIRM to enhance clinical awareness and discuss current knowledge on its diagnosis and management.

## Case presentation

### Patient information

A 10-year-old boy was admitted with a 5-day history of fever and rash. His illness began with a high fever (maximum temperature 39.5°C), cough, and sputum production. Two days later, blistering of the lips developed and gradually eroded. Shortly thereafter, multiple erythematous lesions, some targetoid with small vesicles, appeared on his hands, trunk, and limbs. Initial laboratory tests from a local hospital revealed a white blood cell count of 10.60 × 10^9/L (73.8% neutrophils, 15.4% lymphocytes), and a C-reactive protein level of 27.6 mg/L. Following an inadequate response to empiric antibiotic treatment, he was transferred to our institution.

### Clinical findings

On admission, the patient’s temperature was 38.5°C. He appeared lethargic. Physical examination revealed multiple red macules of varying sizes on the trunk and extremities, some with targetoid morphology and small vesicles. Ocular examination revealed significant conjunctival injection with abundant yellowish secretions. The oral mucosa displayed extensive erosions with limited mouth opening, drooling, and marked pain, while pharyngeal erythema was evident. Lungs had coarse breath sounds without rales; cardiovascular and abdominal examinations were unremarkable.

### Diagnostic assessment

Laboratory evaluation showed:

- White blood cell count: 9.88 × 10^9/L with 75.4% neutrophils.
- Red blood cell count: 4.89 × 10^12/L, Hemoglobin: 145 g/L.
- Platelet count: 280 × 10^9/L.
- C-reactive protein: 17.02 mg/L.
- Interleukin-6: 9.08 pg./mL; total IgE: 28.7 IU/mL; ESR: 95 mm/h; ferritin: 208.37 ng/mL.
- Coagulation: Prothrombin time 15.9 s; fibrinogen 5.58 g/L; D-dimer 1.38 mg/L.
- Liver and renal functions as well as electrolytes were normal.

*M. pneumoniae* nucleic acid testing was performed using multiplex PCR, which was positive. This multiplex PCR method was chosen due to its efficacy in detecting multiple pathogens simultaneously, which is critical for accurate diagnosis in complex cases like MIRM. Comprehensive antinuclear antibody spectrum tests were conducted to rule out autoimmune diseases, including tests for ANA indirect immunofluorescence typing and specific antibodies such as anti-nRNP, anti-SM, anti-SSA, and others, all of which were negative. Additionally, given the patient’s age and lack of relevant history, syphilis was considered highly unlikely, and thus, tests for syphilis were not performed. Our institution does not offer Mycoplasma serology testing; therefore, diagnosis relied solely on nucleic acid detection methods. Serologic assays for herpes simplex virus type I (IgG positive, IgM negative) and type II (both negative), echovirus, coxsackie virus, and enterovirus markers were unremarkable. Blood and throat swab cultures were negative. To further clarify the differential diagnosis, a comparative table is provided below, distinguishing Mycoplasma Induced Rash and Mucositis (MIRM) from Erythema Multiforme (EM), Stevens-Johnson Syndrome (SJS), and Toxic Epidermal Necrolysis (TEN) based on mucocutaneous involvement, triggering pathogens, and severity (Table 1). Electrocardiography indicated sinus rhythm and the chest radiograph did not reveal active pulmonary lesions.

**Table 1:** Comparative table: differential diagnosis of MIRM vs. EM, SJS, TEN.

Condition	Mucocutaneous involvement	Triggering pathogens	Severity
MIRM (mycoplasma induced rash and mucositis)	Predominantly mucosal with some skin lesions	Primarily *Mycoplasma pneumoniae*	Usually moderate, rarely life-threatening
EM (erythema multiforme)	Target lesions, symmetrical distribution on extremities	HSV, Mycoplasma, drugs	Mild to moderate, usually self-limiting
SJS (Stevens-Johnson syndrome)	Mucosal severity less than skin, widespread blisters	Drugs, infections (e.g., Mycoplasma, HSV)	Severe, life-threatening with significant morbidity
TEN (toxic epidermal necrolysis)	Extensive skin detachment (>30% body surface area)	Mainly drugs	Very severe, life-threatening with high mortality

### Diagnosis

**Figure 1:**
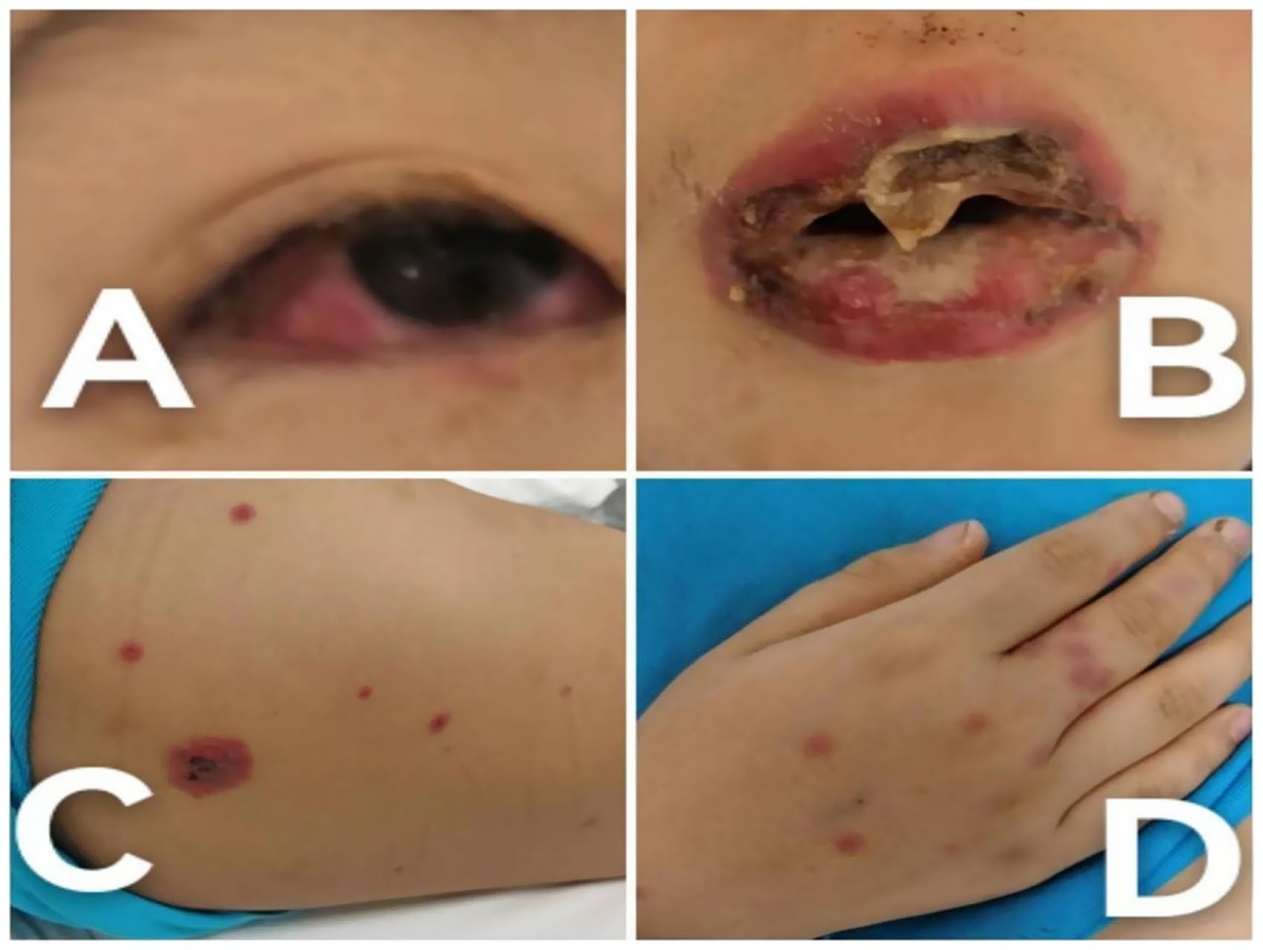
Clinical photographs of the patient at the time of admission. **(A)** Conjunctival hyperemia accompanied by secretions, illustrating the initial severe inflammatory response. **(B)** Severe oral mucosal ulceration with mucosal sloughing, highlighting the acute stage of mucocutaneous involvement. **(C)** Truncal targetoid lesions, depicting the characteristic rash associated with MIRM. **(D)** Hand lesions with a targetoid appearance, demonstrating the extent of skin involvement.

Based on the clinical presentation—limited cutaneous involvement (<10% body surface area) (Figure 1) with prominent mucosal lesions of the eyes and oral cavity—and the positive laboratory evidence for *M. pneumoniae*, a diagnosis of MIRM was established.

### Treatment and outcome

Treatment initially included azithromycin 0.44 g once daily for 5 days, accompanied by methylprednisolone at a dosage of 1 mg/kg/day, and intravenous immunoglobulin (IVIG) administered as 10 g daily for 2 days, followed by 5 g daily for 4 days. Topical management consisted of vitamin C and compound chlorhexidine solution application to the lips and oral mucosa, erythromycin ophthalmic ointment for the eyes, and external agents on the skin lesions. Oral antihistamines (cetirizine and desloratadine) were also prescribed to manage symptoms.

Three days post-treatment, the fever subsided and skin lesions began to resolve, prompting a gradual taper of methylprednisolone. Initially administered at 40 mg daily for 3 days, the dose was then reduced to 30 mg daily for 5 days, and further tapered to 20 mg daily for 7 days. However, after 1 week, despite the initial treatment, the patient’s persistent cough and positive *M. pneumoniae* nucleic acid test indicated an incomplete response to azithromycin. This necessitated an adjustment in the treatment regimen, leading to a switch to oral doxycycline 0.1 g every 12 h for 7 days. The choice of doxycycline over other macrolides or alternatives was based on its proven efficacy in treating *M. pneumoniae* infections, particularly in cases showing resistance to first-line treatments like azithromycin. Additionally, doxycycline is known for its good penetration into respiratory tissues, which is crucial for treating persistent pulmonary symptoms. This decision also considered the growing concern over antimicrobial resistance; doxycycline offers a broader spectrum against potential co-infections and is generally well-tolerated in pediatric patients, making it an appropriate choice in this context. The therapeutic regimen was aimed at preventing long-term sequelae such as persistent mucosal scarring and ocular complications, which are common in untreated or improperly managed cases of MIRM.

**Figure 2:**
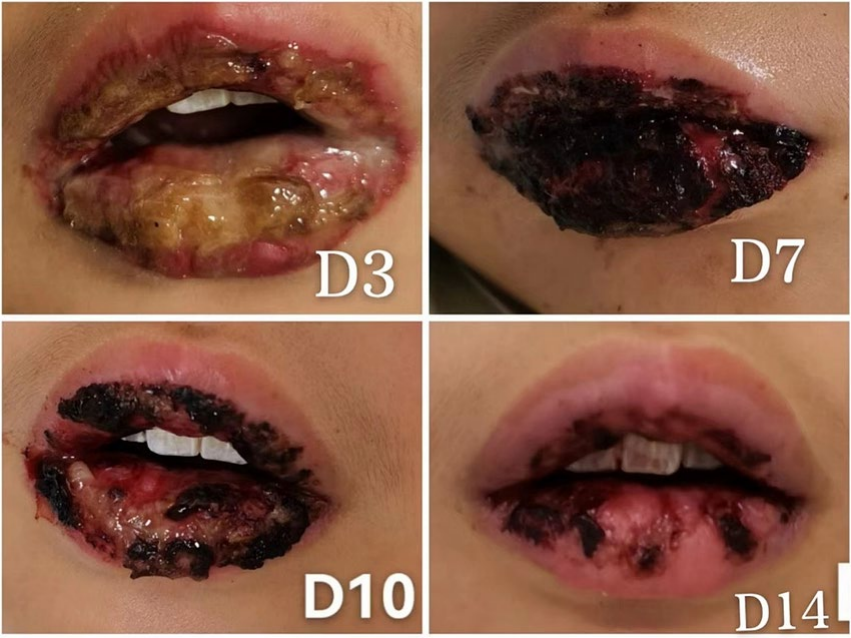
Sequential images showing the healing progression of oral mucosal lesions post-treatment, emphasizing the effectiveness of early intervention. **(D3)** Day 3 post-admission, expansion of oral mucosal ulcerations with ongoing mucosal repair, indicating the initial response to treatment. **(D7)** Day 7 post-admission, oral lesions covered with blood crusts, showing signs of healing beneath the crusts. **(D10)** Day 10 post-admission, partial shedding of the blood crusts revealing the healing mucosa underneath. **(D14)** Day 14 post-admission, most of the oral mucosal has healed with residual crusting, demonstrating significant recovery.

After 14 days of hospitalization, the skin lesions had markedly improved, conjunctival signs resolved, and oral mucosal erosions exhibited crusting and healing (Figure 2). Repeat testing confirmed pathogen clearance and normalization of inflammatory markers, allowing for discharge.

## Discussion

The recognition of *Mycoplasma pneumoniae*-induced rash and mucositis (MIRM) as a distinct clinical entity, separate from other severe mucocutaneous syndromes such as erythema multiforme (EM), Stevens-Johnson syndrome (SJS), and toxic epidermal necrolysis (TEN), highlights significant advances in our understanding of infectious immunopathology (4, 7). MIRM is primarily characterized by significant mucosal involvement with limited skin manifestations, often preceded by respiratory symptoms that serve as crucial diagnostic clues (8).

Recent studies elucidate the pathogenesis of MIRM as involving complex immune-mediated mechanisms rather than simple antibody-mediated reactions (9). It is hypothesized that cytokine dysregulation and T-cell mediated cytotoxicity play pivotal roles in causing the characteristic mucosal damage observed in MIRM (9, 10). Specific studies identify cytokines such as interleukin-6 (IL-6), tumor necrosis factor-alpha (TNF-*α*), and interferon-gamma (IFN-*γ*) as key mediators in this pathogenic process. These cytokines contribute to the T-cell mediated cytotoxicity, further exacerbating the mucosal damage seen in MIRM. The unique cytokine profile, which is distinct from those observed in SJS/TEN, not only aids in distinguishing MIRM clinically but also provides targets for therapeutic intervention, suggesting potential diagnostic markers and therapeutic targets (10).

Reviewing global cases of pediatric MIRM helps in contextualizing the current case within broader epidemiological and clinical patterns. As summarized in the Literature Summary Table, treatments vary widely, with macrolides like azithromycin being common, but outcomes like those reported by Li et al. (11) in Canada and Chowdhury et al. (12) in Singapore show that additional treatments such as immunoglobulins and doxycycline can influence recovery significantly. This variability underscores the importance of tailored treatment approaches based on regional medical practices and antimicrobial resistance patterns (Table 2).

**Table 2:** A summary of cases diagnosed as MIRM in the literature.

Patient age	Patient sex	Systemic features	Mucocutaneous features	Treatment	Outcome	Location	Reference
4	Male	Fever, cough, wheezing	Mucositis, erythematous papules with central hemorrhagic crusts as well as targetoid papulovesicles as rash, conjunctivitis	Cyclosporine, azithromycin, supportive care	Near complete resolution after 7 hospital days	Canada	Li et al. (11)
6	Male	Fever, cough, rhinorrhea, atypical pneumonia	Severe mucositis (oral and penile), small blisters on arms and legs, conjunctivitis	Clarithromycin	Full recovery after 1 week	Singapore	Chowdhury (12)
8	Male	Fever, cough, ill-defined opacity on chest x-ray, leukocytosis	Oral erosions, erythema and swelling of penis, scattered erythematous papules and targetoid lesions, conjunctivitis	Ocular care, supportive care, IVIG, clarithromycin	Improved and discharged in 8 days	Portugal	Santos et al. (21)
8	Female	Cough and dyspnea 1 week prior, fever	Oral, ocular and vulvar mucositis, mild rash <1% BSA (some atypical targetoid papules)	Levofloxacin, IVIG, supportive care	Gradual improvement followed by recurrence of mucositis 9 months later with positive *Mycoplasma pneumoniae* and 2 years later with influenza B	USA	Mazori et al. (22)
8	Female	Fever, cough	conjunctivitis in both eyes and ulcers in the oropharynx, conjunctivitis, oropharynx ulcers, vesicular lesions, Targetoid lesions, vulvar and perianal regions ulcers	doxycycline	Recover after 2 weeks	China	Lu et al. (23)
9	Male	Fever, cough, tachypneic	conjunctivae, oropharynx ulcers	Azithromycin, intravenous vancomycin, parenteral nutrition, supportive care	Complete recovery after a 33-day hospitalization in good condition and without permanent sequelae	USA	Valle et al. (15)
10	Male	Self-limited fever and cough 1 week prior	Ulcerative and hemorrhagic oral ulcers, mild serpiginous eruption on skin	Systemic steroids, clarithromycin, supportive care	Complete resolution in 1 week	Italy	Poddighe et al. (24)
11	Male	Cough, Low grade fever, atypical pneumonia	Oral ulcers, conjunctivitis, hemorrhagic crusts of nasal mucosa, genital skin sloughing, no rash	Azithromycin, systemic steroids, supportive care	Not noted	USA	Bowling et al. (25)
12	Female	Fever, sore throat, cough	Oral lesions with mucosal sloughing, conjunctivitis, vesicular rash	Azithromycin, acyclovir, IVIG, supportive care	Recovered and re-presented 3 years later with similar symptoms and PCR positive for Chlamydophila pneumoniae	USA	Brazel et al. (26)
13	Male	Conjunctivitis, pustules, targetoid lesions, pruritis, burning, macules on buccal mucosa	Erosive oral and genital lesions, conjunctivitis, no cutaneous lesions	Systemic and topical corticosteroids, doxycycline	Significant improvement within 2 days of doxycycline	USA	Chao et al. (27)
13	Female	Cough, rhinitis	Erosive oral and genital lesions, conjunctivitis, no cutaneous lesions	Azithromycin later changed to doxycycline, cyclosporine, supportive care	Had a past history of MIRM before this episode, near complete resolution after 6 days of treatment	Canada	Li et al. (11)
14	Female	Cough, sore throat, dyspnea, diplopia, orthopnea, malaise, headache, nausea, vomiting	Oral and vaginal erosions, conjunctivitis	Cyclosporine, azithromycin, supportive care	Complete resolution of oral lesions by 1 week after hospital discharge	Canada	Li et al. (11)
15	Male	Fever, decreased appetite 10 days prior, cough, diarrhea, atypical opacities on chest x-ray	Hemorrhagic conjunctivitis, diffuse oral and pharyngeal erosions, crust at urethra, rare papulovesicles on skin	Azithromycin, supportive care	Gradual improvement over days	USA	Curtiss et al. (28)
15	Male	Fever, dry cough	Conjunctivitis, oral erosions, targetoid papules and vesicles on skin	Azithromycin, IVIG	Slow improvement over 1 month with recurrence that resolved with azithromycin, postinflammatory hyperpigmentation	USA	Song et al. (29)
18	Male	Fever, sore throat, cough	Conjunctival injection, targetoid vesicles on face, torso, and penis, and mucositis over lips and buccal mucosa	Azithromycin, systemic prednisone	Azithromycin, systemic prednisone	USA	Sandhu et al. (30)
20	Male	Fever, leukocytosis	Conjunctivitis, oral ulcerations	Azithromycin, IV methylprednisolone transitioned to oral, supportive care	Full recovery in 10 days	USA	Kheiri et al. (31)
33	Male	Fever, productive cough, myalgias, nasal congestion, sore throat	Oral ulcers on the lips, vesicular rash, penile meatus ulceration	Azithromycin, methylprednisolone, supportive care	Recover after a month	USA	Gandelman et al. (10)
42	Male	Upper respiratory illness	Conjunctivitis, oral and genital erosions, atypical targetoid papules and plaques	Azithromycin, supportive care	Previous history of similar illness as a child, resolution after 1 month with continued oral pain and phimosis	USA	Song et al. (29)
46	Male	Atypical pneumonia	Mucositis, conjunctivitis	Azithromycin, systemic corticosteroids, supportive care, gentamicin/ dexamethasone eye drops	Complete recovery	Portugal	Zão et al. (32)

BSA, body surface area; IV, intravenous; IVIG, intravenous immunoglobulin; MIRM, M pneumoniae-induced rash and mucositis; PCR, polymerase chain reaction.

The treatment strategies for MIRM remain largely empirical; however, emerging data suggests that early intervention with targeted therapies such as antibiotics against *Mycoplasma pneumoniae*, corticosteroids, and intravenous immunoglobulin can significantly modify the disease course (2, 13). These treatments not only reduce the duration of mucosal involvement but also minimize the risk of long-term sequelae. Additionally, the use of cyclosporine A has shown promise in rapidly alleviating symptoms and reducing hospital stays in severe cases. Cyclosporin A, a potent immunosuppressant, inhibits calcineurin, thereby reducing T-cell activation and cytokine release. This mechanism is particularly beneficial in controlling the inflammatory responses observed in severe cases of MIRM, where traditional treatments might be insufficient. This suggests new avenues for therapeutic approaches that could be explored in future clinical trials (11).

The prognosis of MIRM is generally favorable compared to the often grave outcomes associated with SJS and TEN. However, the risk of recurrence and long-term complications such as ocular complications or oral scarring remains a concern (14). Chronic complications like conjunctival scarring and dry eye syndrome may develop, underscoring the importance of vigilant ophthalmologic evaluation and a multidisciplinary approach to care, which should integrate dermatologic, ophthalmologic, and infectious disease expertise (14, 15).

Additionally, recent findings suggest that genetic predispositions, including specific HLA haplotypes, may influence individual immune responses to *M. pneumoniae* infection, potentially accounting for the variability in clinical presentation and severity observed in MIRM (16–20). This insight not only deepens our understanding of the disease mechanism but may also pave the way for more personalized therapeutic strategies.

In summary, this case report contributes novel insights into the management of *Mycoplasma pneumoniae*-induced rash and mucositis (MIRM), highlighting a unique clinical presentation and therapeutic response. Notably, this case from China illustrates a robust response to doxycycline, diverging from typical treatment approaches and suggesting regional variations in medical practice that might influence treatment efficacy. This supports the need for a dynamic model of MIRM, recognizing it as distinct from other severe mucocutaneous syndromes such as EM, SJS, and TEN. Early recognition and aggressive management tailored to specific clinical contexts are crucial to prevent complications and improve outcomes. The integration of emerging immunomodulatory therapies, including the successful use of doxycycline in this instance, into standard care protocols, underscores the potential to significantly enhance patient care and adapt treatment strategies to regional medical practices.

## Strengths and limitations

This report has several strengths that contribute to the understanding of *Mycoplasma pneumoniae*-induced rash and mucositis (MIRM). Firstly, it draws from a comprehensive review of literature, incorporating findings from recent systematic studies, which enhances the depth and relevance of the discussion. Secondly, the inclusion of a pediatric case provides practical insights into the clinical presentation and management of MIRM, adding to the real-world applicability of the information. Thirdly, the discussion integrates emerging research on the pathogenesis and potential genetic predispositions involved in MIRM, offering a forward-looking perspective that could influence future therapeutic approaches.

However, there are several limitations to this study. The report is based on a single case study, which may not fully represent the broader spectrum of clinical presentations and outcomes associated with MIRM. This limits the generalizability of the findings. Additionally, potential diagnostic misclassification must be acknowledged, as similar symptoms can occur in other mucocutaneous diseases such as EM, SJS, and TEN, which might lead to diagnostic uncertainty. The absence of comprehensive serologic testing (e.g., lack of IgM/IgG trending) further complicates the accuracy of pathogen identification, possibly affecting the diagnosis and understanding of the immune response. Moreover, while the report discusses potential new treatments such as the use of cyclosporine A, these are based on limited case reports and lack robust evidence from controlled clinical trials. Thus, the efficacy and safety of these treatments remain to be validated in larger, more diverse populations. Furthermore, the discussion on genetic factors influencing MIRM is based on preliminary data, which requires further investigation to confirm these associations and understand their clinical implications. Lastly, as MIRM is a relatively newly recognized condition, long-term follow-up data on patients is scarce, which constrains our understanding of the chronic sequelae and long-term prognosis of the disease. Additionally, there is variability in therapeutic response among individuals, which could influence the efficacy and outcomes of the treatments administered. This factor should be considered when interpreting the results and applying them to broader clinical practice.

## Take-away lessons

*Mycoplasma pneumoniae*-induced rash and mucositis (MIRM) stands as a distinct clinical entity that necessitates early recognition due to its unique presentation, which can often be mistaken for other mucocutaneous syndromes. This differentiation is crucial as it informs the tailored clinical approach required for MIRM, which includes the use of specific antibiotics, corticosteroids, and possibly immunoglobulin therapies aimed at reducing symptom severity and duration. Emerging therapeutic strategies, such as the use of cyclosporine A, highlight the evolving nature of treatment protocols, though these require further validation through robust clinical trials. Additionally, preliminary findings suggest genetic factors may influence individual responses to *Mycoplasma pneumoniae*, pointing towards the potential for personalized medical approaches in the future. Ongoing research is essential to solidify these findings and to explore the long-term outcomes of MIRM, ensuring that treatment strategies continue to improve and are based on strong scientific evidence.

## Patient perspective

Navigating *Mycoplasma pneumoniae*-induced rash and mucositis (MIRM) poses both physical and emotional challenges for patients and their families. Initially, the onset of respiratory symptoms followed by severe and painful mucosal lesions can be deeply distressing, often leading to significant discomfort and anxiety. The rarity and complexity of MIRM can also cause delays in diagnosis, exacerbating the uncertainty and stress associated with managing this condition.

From a patient’s viewpoint, understanding the illness is crucial. Effective communication with healthcare providers ensures that patients and their families are well-informed about the illness, treatment options, and prognosis. This knowledge is vital for managing symptoms at home and preparing for potential hospital visits or treatments. Additionally, access to psychological support and connecting with others who have faced similar health challenges can provide much-needed emotional support and reduce feelings of isolation.

## Conclusion

A timely and accurate diagnosis of MIRM is crucial for instituting appropriate treatment and minimizing morbidity. A multidisciplinary approach, combining antimicrobial therapy with judicious use of corticosteroids, IVIG, and possibly other immunomodulatory agents, has the potential to improve outcomes (18–20). Ongoing research into the immunogenetic underpinnings of MIRM may ultimately enable more individualized treatment strategies and further reduce the risk of long-term sequelae (16–20).

## Data Availability

The raw data supporting the conclusions of this article will be made available by the authors, without undue reservation.
